# Expression of Concern: Can digital transformation promote green innovation in enterprises? the moderating effect of heterogeneous environmental regulations

**DOI:** 10.1371/journal.pone.0337272

**Published:** 2025-11-21

**Authors:** The PLOS One Editors

Contrary to the declaration in this article’s [1] Data Availability statement, the individual-level underlying data supporting the article’s results were not provided. As such, this article [1] does not comply with the PLOS Data Availability policy.

In addition, the *PLOS One* Editors have concerns about the article’s peer review.

In light of the cumulative issues, the *PLOS One* Editors issue this Expression of Concern. Readers are advised to interpret the article [1] with caution.
