# Supplementary material for: Can digital transformation promote green innovation in enterprises? the moderating effect of heterogeneous environmental regulations
Source: PLoS One. 2024 Jun 6;19(6):e0298191. doi: 10.1371/journal.pone.0298191 (PMC11156353; doi:10.1371/journal.pone.0298191)
Supplement: Attachment — Submitted filename: Response to Reviewers.docx [file pone.0298191.s001.docx]

**Journal Requirements**

When submitting your revision, we need you to address these additional requirements.

1. Please ensure that your manuscript meets PLOS ONE's style requirements, including those for file naming. The PLOS ONE style templates can be found at

https://journals.plos.org/plosone/s/file?id=wjVg/PLOSOne_formatting_sample_main_body.pdf and

<https://journals.plos.org/plosone/s/file?id=ba62/PLOSOne_formatting_sample_title_authors_affiliations.pdf>

**Response: Thanks for your comments. Modifications have been made in the article.**

2. Thank you for stating the following financial disclosure:

~~"Supported by The Project of Cultivation for young top-motch Talents of Beijing Municipal Institutions (BPHR202203211), Innovation Centre for Digital Business and Capital Development of Beijing Technology and Business University (SZSK202237) and Academic Research Projects of Beijing Union University (SK10202201)."~~

**Response: Thanks for your comments. This study was funded by the General Project of the Beijing Social Science Foundation (23GLB020).**

Please state what role the funders took in the study. If the funders had no role, please state: "The funders had no role in study design, data collection and analysis, decision to publish, or preparation of the manuscript."

If this statement is not correct you must amend it as needed.

Please include this amended Role of Funder statement in your cover letter; we will change the online submission form on your behalf.

**The sponsor of the article is the corresponding author of the article**

PLOS requires an ORCID iD for the corresponding author.

4. In the online submission form, you indicated that "The datasets used and/or analysed during the current study available from the corresponding author on reasonable request."

All PLOS journals now require all data underlying the findings described in their manuscript to be freely available to other researchers, either 1. In a public repository, 2. Within the manuscript itself, or 3. Uploaded as supplementary information.

This policy applies to all data except where public deposition would breach compliance with the protocol approved by your research ethics board. If your data cannot be made publicly available for ethical or legal reasons (e.g., public availability would compromise patient privacy), please explain your reasons on resubmission and your exemption request will be escalated for approval.

**We selected Chinese manufacturing listed companies from 2010 to 2021 as our research sample, which involves official data from many companies. We only conducted statistical analysis on the data, and the companies did not authorize the data, so it is inconvenient to provide it. Please understand!**

**Additional Editor Comments:**

1. The theoretical contributions could be better articulated, linking them back to the identified conversants and the research methods employed. This, in tandem with an explicit depiction of how the results impact our understanding of the research topic, would substantially elevate the study's contribution. It would be beneficial if the authors could identify the papers they aim to contribute to and delineate their novel addition to the existing discourse.

**Response: Thanks for your comments. Revised in the article, detailed in sections 5.1-5.4, conclusion and outlook.**

2. It might be helpful with new citations of up-to-date and high-quality papers on the subjects covered in manuscript and engaged the previous literature in this journal.

**Response: Thanks for your comments. Journal articles were cited in references 20, 24, and 29.**

3. Also, the author(s) should carefully format the manuscript following the guidance of the journal, such as the headers, references, and so on. There are mistakes in the citation (reference), such as missing volume, issue and page.

**Response: Thanks for your comments. The article has been extensively revised.**

4. The authors are recommended to examine and proof the language. In addition, there are some typos and grammatical errors that need further attention.

**Response: Thanks for your comments. The article has been extensively revised.**

**Reviewers' comments:**

**Comments to the Author**

Reviewer #1:

This paper has a novel research theme and provides an important reference for the implementation of enterprise digital transformation strategy. In addition, this paper has made a solid review of the existing literature, and the theoretical analysis is clear and direct, but there are several contents that can be optimized as follows.

1. The content of the revelation part of the paper is repeated, so it is suggested to sort it out again.

**Response: Thanks for your comments. The conclusion and presentation have been rephrased, and duplicate content has been removed.**

2. At present, the marginal contribution of the article is too much, please briefly present the marginal contribution of the article.

**Response: Thanks for your comments. The marginal contribution is elaborated in the article, which is reflected in sections 5.2, 5.3, 5.4, and conclusion.**

Reviewer #2:

Digital transformation is an important way for enterprises to achieve green innovation. This paper takes listed manufacturing companies in China as samples, empirically tests the impact of digital transformation on enterprise green innovation and its intermediate mechanism, and expands the relevant research on digital transformation. However, this article would be more complete if it could be improved in the following aspects.

1. Supplement the chapter arrangement of the paper at the end of the introduction.

**Response: Thanks for your comments. The concluding part of the introduction has been modified.**

2. Make clear the theoretical contribution of this paper compared with existing research.

**Response: Thanks for your comments. The conclusion and presentation have been rephrased and discussed in detail.**

3.The writing specification of the article should be further matched with the publication requirements，such as author format and references.

**Response: Thanks for your comments. Modifications have been made in the article.**
